# Brainstem Neurons Survive the Identical Ischemic Stress That Kills Higher Neurons: Insight to the Persistent Vegetative State

**DOI:** 10.1371/journal.pone.0096585

**Published:** 2014-05-06

**Authors:** C. Devin Brisson, Yi-Ting Hsieh, Danielle Kim, Albert Y. Jin, R. David Andrew

**Affiliations:** Department of Biomedical & Molecular Sciences, Queen's University, Kingston, Ontario, Canada; University Hospital-Eppendorf, Germany

## Abstract

Global ischemia caused by heart attack, pulmonary failure, near-drowning or traumatic brain injury often damages the higher brain but not the brainstem, leading to a ‘persistent vegetative state’ where the patient is awake but not aware. Approximately 30,000 U.S. patients are held captive in this condition but not a single research study has addressed how the lower brain is preferentially protected in these people. In the higher brain, ischemia elicits a profound anoxic depolarization (AD) causing neuronal dysfunction and vasoconstriction within minutes. Might brainstem nuclei generate less damaging AD and so be more resilient? Here we compared resistance to acute injury induced from simulated ischemia by ‘higher’ hippocampal and striatal neurons versus brainstem neurons in live slices from rat and mouse. Light transmittance (LT) imaging in response to 10 minutes of oxygen/glucose deprivation (OGD) revealed immediate and acutely damaging AD propagating through gray matter of neocortex, hippocampus, striatum, thalamus and cerebellar cortex. In adjacent brainstem nuclei, OGD-evoked AD caused little tissue injury. Whole-cell patch recordings from hippocampal and striatal neurons under OGD revealed sudden membrane potential loss that did not recover. In contrast brainstem neurons from locus ceruleus and mesencephalic nucleus as well as from sensory and motor nuclei only slowly depolarized and then repolarized post-OGD. Two-photon microscopy confirmed non-recoverable swelling and dendritic beading of hippocampal neurons during OGD, while mesencephalic neurons in midbrain appeared uninjured. All of the above responses were mimicked by bath exposure to 100 µM ouabain which inhibits the Na^+^/K^+^ pump or to 1–10 nM palytoxin which converts the pump into an open cationic channel.

Therefore during ischemia the Na^+^/K^+^ pump of higher neurons fails quickly and extensively compared to naturally resilient hypothalamic and brainstem neurons. The selective survival of lower brain regions that maintain vital functions will support the persistent vegetative state.

## Introduction

The persistent vegetative state evolves from hypoxic-ischemic encephalopathy that involves the entire brain and is caused by cardiac/pulmonary arrest, strangulation or near drowning. As well brain swelling secondary to traumatic brain injury is a common cause of global ischemia. In 1994 10,000 to 25,000 U.S. adults and 4,000 to 10,000 children were captive in this state of being “awake but not aware” [1], [2]. With profound damage to higher brain regions, the hypothalamus and brainstem somehow survive to sustain life [3]–[5] as demonstrated by brain imaging [6]–[9] and regional brain metabolism measurements [10]. This rostral vulnerability to ischemia is also apparent in animal models of global ischemia [11]–[13]. Given that the brain is globally deprived of blood, what makes the brainstem so resilient? No study has addressed that question to date.

Within about a minute of the onset of global ischemia [14], [15] or stroke [16]–[18], synapses initially fail and then a profound ‘ischemic’ or ‘anoxic’ depolarization (AD) propagates as a front of neuronal inactivation and swelling across the neocortex. Synaptic silence and then AD result from failure of the Na^+^/K^+^ pump and any recovery requires energy from the already compromised gray matter. This causes acute injury to the neurons within minutes if blood flow does not return. During 10 minutes of oxygen/glucose deprivation (OGD) in live brain slices, neocortex [19], [20], hippocampus [21], [22], striatum [13], thalamus [23], [24] and cerebellar cortex [25] undergo AD that then acutely injures the resident neurons over several minutes. The extent of AD reliably determines ensuing neuronal damage in higher brain [18]. In contrast, ‘lower’ neurons in hypothalamus generate a weak version of AD in vivo [11] and resist acute injury caused by OGD [24], [26]. Previous work has shown that, perhaps like hypothalamus, some brainstem nuclei of adult rat do not support strong spreading depolarization [27], [28] unless chemically depolarized [29]. Thus brainstem neurons might better survive the stress of global ischemia noted above by resisting AD or by better recovering post-AD compared to higher neurons.

We tested this hypothesis by using OGD to stress higher neurons recorded in CA1 pyramidale and in striatum, contrasting their responses and survivability with lower neurons from four brainstem nuclei. We monitored AD using light transmittance (LT) imaging, whole-cell patch recording and 2-photon laser scanning microscopy in coronal brain slices of adult rat exposed to 10 min of simulated ischemia. The use of slices ruled out that variable regional blood flow influences outcome. We show that brainstem neurons have the intrinsic ability to resist shutdown by AD. By maintaining resting potential, avoiding spike inactivation and rapidly recovering from low-oxygen/low-glucose, brainstem circuitry is better preserved. We propose that this sustains vital bodily functions even as higher brain regions deteriorate, characteristic of the persistent vegetative state.

## Materials and Methods

### Brain Slice Preparation

All animal care and use was conducted in accordance with the national standards set down by the Canadian Council for Animal Care. Sprague-Dawley rats (21–60 days old; Charles River, St. Constant, PQ) or Wistar-YFP mice (40–100 days old; bred in-house) were decapitated by guillotine under a protocol which included housing standards that was approved by the Queen's University Animal Care Committee. Following craniotomy, the olfactory bulbs and optic tracts were cut. The brain was quickly removed and immersed in ice-cold and oxygenated (95% O_2_, 5% CO_2_) artificial cerebral spinal fluid (aCSF) composed of (in mM) 240 sucrose, 3.3 KCl, 26 NaHCO_3_, 1.3 MgSO_4_.7H_2_O, 1.23 NaH_2_PO_4_, 11 D-glucose and 1.8 CaCl_2_. Using a Leica 1000-T vibratome, 400 µm slices were cut in the sucrose aCSF, usually in the coronal plane. Slices were incubated in regular aCSF (equimolar NaCl replacing sucrose above) at 35°C for at least 1 hour. Slices were then transferred to a recording/imaging chamber where they were submerged in flowing aCSF (3 ml/min.) at 36°C±0.5°C. The aCSF osmolality was 295 mOsm at pH 7.4. Following exposure to 10 min of OGD, 100 µM ouabain or 1–10 nM palytoxin, slices were reintroduced to control aCSF for 30 minutes to observe the extent of recovery.

### Imaging changes in light transmittance (ΔLT)

Slices were imaged using near-infrared illumination through an upright microscope (Axoscope 2FS, Zeiss) with a 40× immersion objective lens. Video images were captured with a cooled charged coupled device (Hamamatsu C4742) using Imaging Workbench 6 software (Indec Biosystems Inc.). Each image of a video series consisted of 16 averaged frames acquired at 20 Hz. The first image of the series was the control transmittance (*T_cont_*) which was subtracted from each of the subsequent images (*T_exp_*) in the series. The difference signal was normalized by dividing by *T_cont_*, which varies across the slice depending on the zone sampled. For example, *T_cont_* was lower in white matter than gray matter. This value was then presented as a percentage of the digital intensity of the control image of that series. That is, ΔLT = [(*T_exp_−T_cont_*)/*T_cont_*]×100 = [ΔT/T]%. The change in LT was displayed using a pseudocolor intensity scale. The slice image in bright field was displayed using a gray intensity scale. In some experiments, single neurons were visualized using near-infrared illumination and Dodt gradient contrast optics (Luigs and Neumann, Ratingen, Germany). Note that the initial swelling response to OGD as imaged by elevated LT signaling involves both neurons and astrocytes while the subsequently reduced LT response indicates injured (beaded) dendrites. Astrocytes quickly recover and do not contribute to the reduced signal [30], [31].

### Electrophysiology

Micropipettes were pulled from borosilicate glass (outside diameter 1.2 mm, inside diameter 0.68 mm; World Precision Instruments) to a resistance of 3–6 MΩ. The internal pipette solution contained (in mM) 125 K- gluconate, 10 KCl, 2 MgCl_2_, 5.5 EGTA, 10 HEPES, 2 Na-ATP and 0.1 CaCl_2_ (pH was adjusted to 7.3 with KOH). A 14 mV junction potential was corrected prior to achieving whole-cell configuration. Visually guided whole-cell patch recordings were obtained in hippocampal CA1 pyramidale, striatum, the mesencephalic nucleus (MES) of midbrain-pons, locus coeruleus (LC) of midbrain-pons, dorsal nucleus solitarius (dNTS) of medulla oblongata and dorsal motor nucleus (DMV) of medulla oblongata. All recordings were acquired in current clamp mode of an Axoclamp 2A amplifier and a Digidata 1322 A/D converter (Axon instruments). Clampex 10 software (Axon instruments) was used for data acquisition with subsequent analysis using Clampfit 10 software. Sampling frequency was 10 kHz and low pass filtering was with an external Bessel filter (LPF 202a; Axon Instruments) at 2 kHz. After obtaining whole-cell recordings, slices were simultaneously imaged (below) while exposed to oxygen glucose deprivation (OGD). The OGD aCSF was of similar composition to control aCSF, except for substituting of 95% O_2_/5% CO_2_ bubbling of aCSF with 95% N_2_/5% CO_2_. In addition, 11 mM glucose was reduced either to 0 mM or 1 mM glucose with osmotic adjustment using NaCl. Occasionally, brainstem neurons were exposed to multiple applications of OGD and/or newly acquired recordings obtained post-OGD in the same slice.

### Two-Photon Laser Scanning Microscopy

Coronal brain slices (400 µm thick) were taken from 30+ day-old C57 black mice of the B6.Cg-Tg (Thy1-YFP)16 Jrs/J strain and were prepared as described for rat slices. The mouse aCSF composition was the same but with 20 mM mannitol added to balance the higher mouse plasma osmolality. These mice have a proportion of pyramidal neurons that express yellow fluorescent protein (YFP) [32] and so the neurons can be imaged in real time during OGD [19], [20], [24], [26], [33], [34]. Structural responses to OGD by mouse slices [30], [34] are similar to the cell swelling and dendritic beading observed in rat slices [35]. An imaging chamber was mounted on a fixed stage of an upright Axioscope II FS microscope (Carl Zeiss, Jena, Germany). YFP^+^ neurons were imaged with appropriate filter sets using a Zeiss LSM 710 NLO meta multiphoton system coupled to a Coherent Ti:Sapphire laser. Three-dimensional image stacks were taken at 3.0-µm increments using a Zeiss 40× or 63× water-immersion objective. Data acquisition and analyses were controlled by Zeiss LSM software.

### Statistical Analysis

Light transmittance data from each brain region of interest (∼500 µm^2^) were averaged and plotted +/− standard error. Means were then compared with an independent samples t-test, with compared distributions first checked for normality using a one-sample Kolmogorov-Smirnov test, all p's>0.05. Whole-cell patched neurons were analyzed if they displayed stable resting membrane potentials and if the series resistance could be sufficiently compensated. Electrophysiological data were presented as means +/− standard deviation.

## Results

### Light Transmittance Imaging

**Figure 1:**
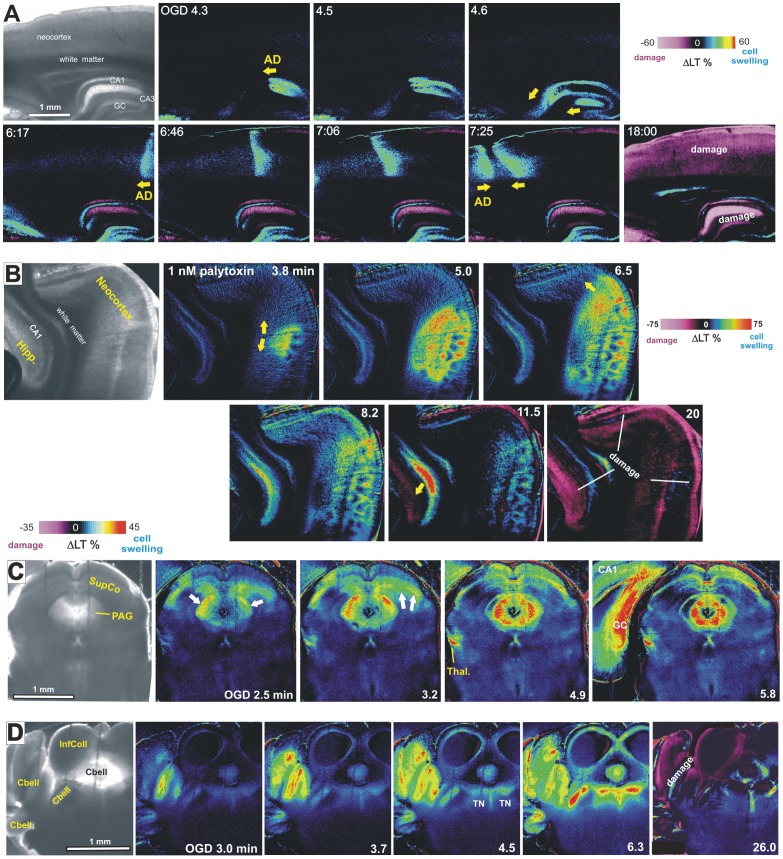
Light transmittance (LT) imaging reveals damage following AD in higher, but not lower, brain regions. A) The front of LT change representing AD onset arises and propagates as distinct waves coursing through hippocampal and neocortical gray matter (arrows) leaving damage (light scatter, purple) in its wake. B) Palytoxin introduced at zero minutes induces OGD-like AD at extremely low concentrations by converting the Na^+^/K^+^ pump to cationic channel. The resulting LT front initiates in neocortex and then hippocampus. Note spreading swelling in the CA1 cell body layer (red). These cortical regions are subsequently damaged (purple). These findings were replicated in 30 of 30 slices using 10 or 1 nM palytoxin. With 0.1 nM palytoxin, AD was observed in 12 of 16 slices but rarely in subcortical regions [71]. C) In midbrain-pons, AD propagates as ‘mini-fronts’ through superior colliculus and PAG, causing swelling but no damage. Superficial superior colliculus is an exception (see Discussion). D) More caudally, AD swells and damages cerebellar gray, but only temporarily swells tegmental nuclei (TN) in the pons.

Imaging elevated LT in brain slices or light reflectance (LR) from the neocortical brain surface reveals cell swelling-associated signals (LT increases or LR decreases) as the front of LT change propagates across gray matter [14], [34]. In the wake of this front, which has been well documented as representing encroaching AD, synaptic spines disappear as dendritic beads form. The beading scatters light thereby decreasing LT and increasing LR. Within 6 minutes of OGD the gray matter of neocortex, hippocampal formation, striatum (Movie S1) and thalamus (not shown) display striking AD onset and propagation. Wherever the AD front spreads (Fig. 1A, arrows), light scatter denoting injury arises within several minutes (Fig. 1A and Movie S1, purple pseudocoloring). This propagating sequence of swelling and then injury is replicated in slices of higher brain regions by inhibiting the Na^+^/K^+^ pump with bath exposure to 100 µM ouabain (Movie S2) or by converting the pump into an open cationic channel using 1–10 nM palytoxin (Fig. 1B). In neocortex, striatum, hippocampus and thalamus either treatment evokes AD-like LT changes identical to OGD. That is, an elevated LT front of cell swelling denoting AD onset gives rise to an irreversible decrease in LT wherever the front has propagated.

**Figure 2:**
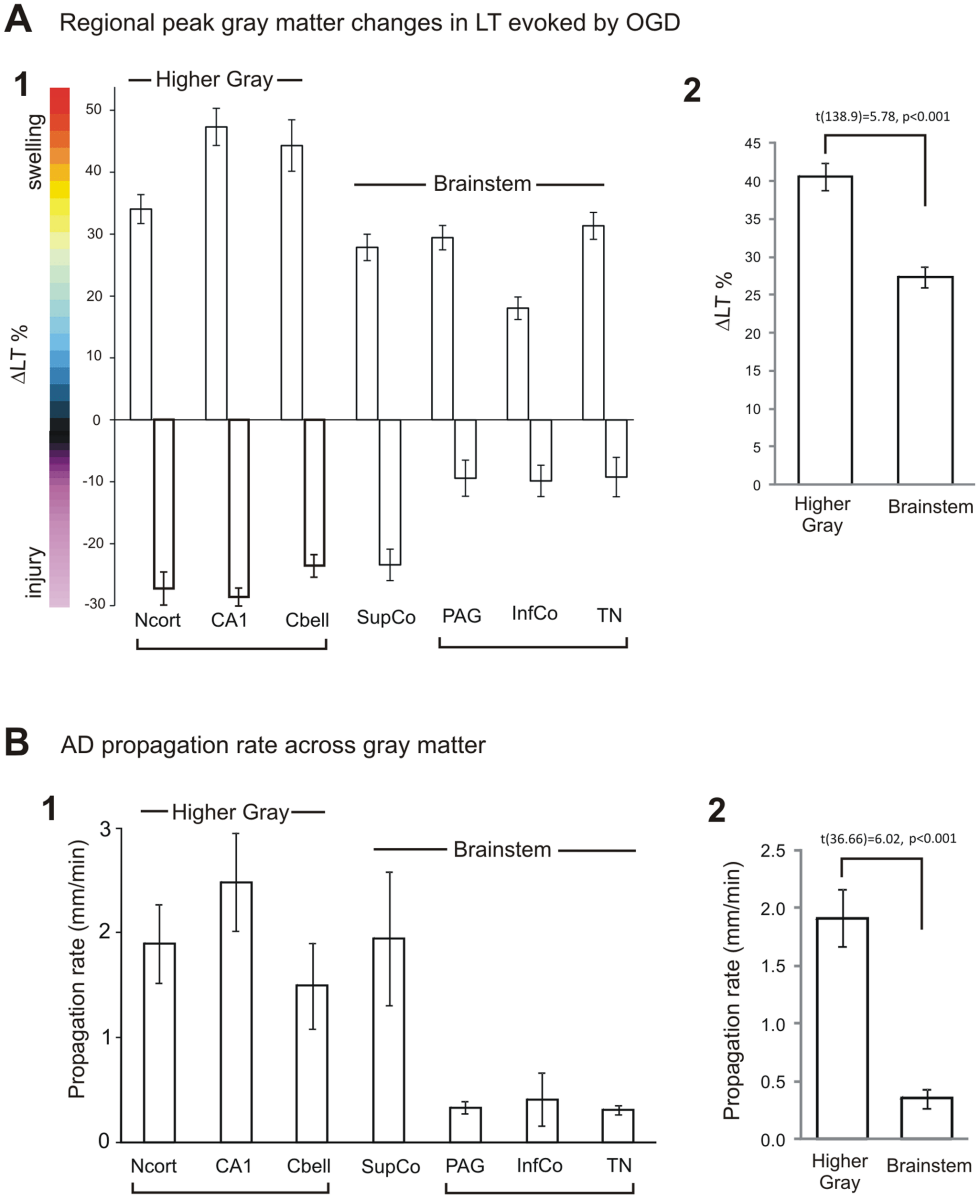
AD generation is stronger in rostral brain regions compared to brainstem. A1) Comparison of regional responses to OGD in terms of AD strength (peak LT increases) and subsequent dendritic injury (maximal LT decreases) shows more swelling and subsequent injury by higher brain regions compared to brainstem nuclei. The exception is superior colliculus, which displays injury similar to higher gray matter, possibly because it has been a prominent surface structure throughout vertebrate evolution until recently. A2) The means of peak LT values displayed by higher and lower regions (excluding SupCo) were collapsed and compared with an independent samples t-test and found to be significantly different [t(58) = 3.6, p<0.001]. B1) Propagation speed of the AD front is faster in higher brain gray matter than brainstem nuclei, again with the exception of superior colliculus. B2). The means of the rate (mm/min) displayed by higher and lower regions (excluding SupCo) were collapsed and compared with an independent samples t-test and found to be significantly different [t(36.7) = 6.02,p<0.001].

In OGD slices containing brainstem, the adjacent hippocampus (Fig. 1C) or cerebellar cortex (Fig. 1D) supports a spreading LT front with subsequent damage. At the same time the brainstem nuclei display OGD-induced ‘mini-fronts’ of AD coursing through the periaqueductal gray, superior colliculus (Figs. 1C, 2A1), inferior colliculus (Fig. 1D, 2A1) and tegmental nucleus (Figs. 1D, 2A1). However these lower brain regions display little subsequent light scatter with the exception of superior colliculus (Fig. 2A1). LT values from the three higher brain areas (neocortex, hippocampus and cerebellar cortex) and three lower brain areas (PAG, IC and TG) were collapsed and the means plotted in Figure 2A2. These means were compared with an independent samples t-test and found to be significantly different [t(138.9) = 5.78, p<0.001]. Similarly the three higher regions showed significantly more LT reduction than the three lower regions (not shown).

The AD propagation rate was calculated in brainstem nuclei and compared to higher gray matter, often in the same brain slices (Fig. 2B). AD propagation rates from the three higher brain areas (neocortex, hippocampus and cerebellar cortex) and three lower brain areas (PAG, IC and TG) were collapsed and the means plotted in Figure 2B2. These means were compared with an independent samples t-test and found to be significantly different [t(36.7) = 6.02,p<0.001; df corrected for unequal variances]. The AD propagation rate appeared more robust in higher brain regions, again with the exception of superior colliculus (see Discussion).

We followed up these observations which indicated less AD-evoked damage in the brainstem post-OGD but using whole-cell patch recording of neurons during OGD to compare responses of higher gray matter with four brainstem nuclei. The electrophysiological properties of each recorded neuron as well as the properties of lower neurons recorded in post-OGD slices can be found at http://hdl.handle.net/1974/7578 [36]. General findings are presented below.

### Higher Neuron Electrophysiology

#### CA1 Pyramidal Cells

**Figure 3:**
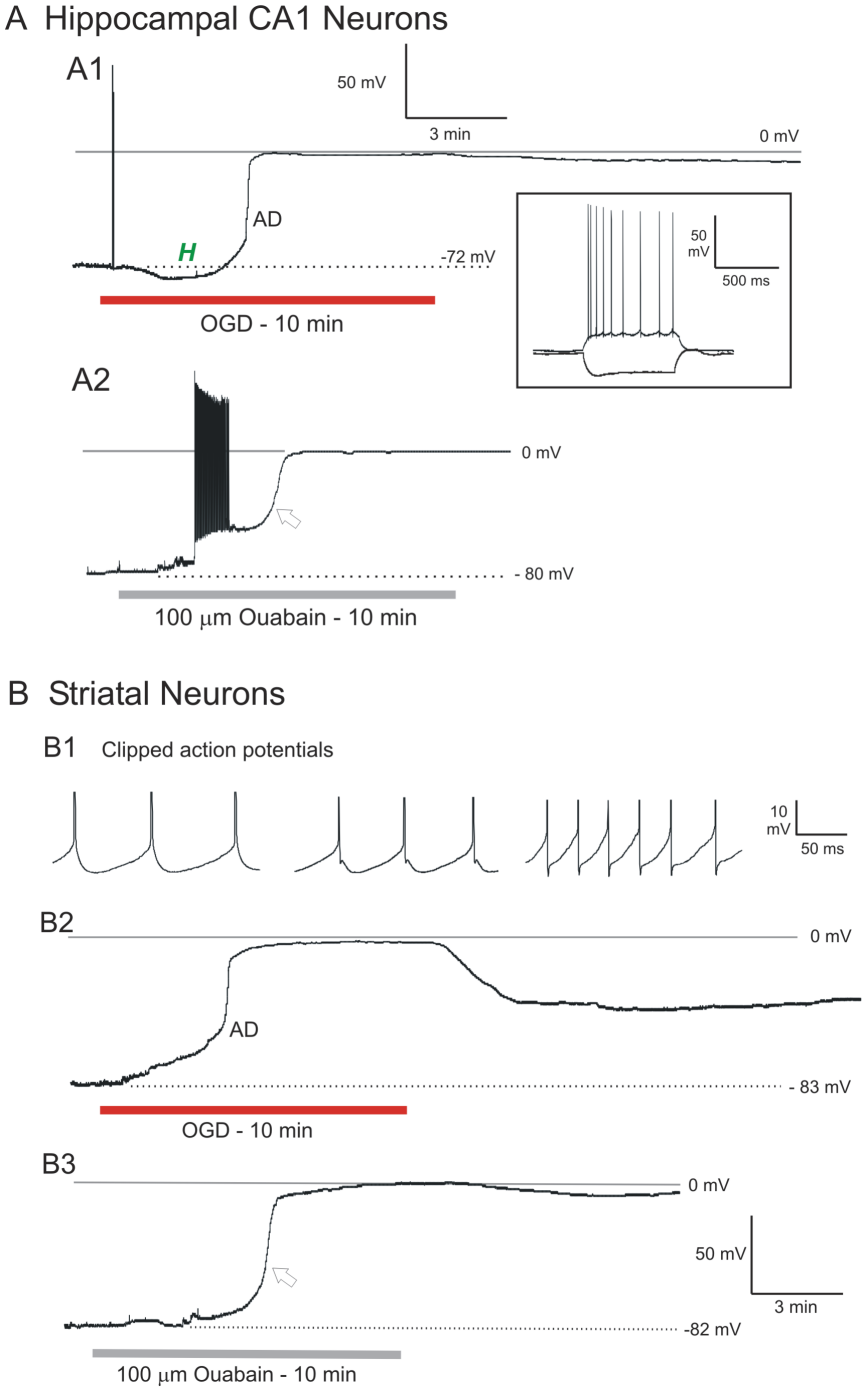
Whole-cell recordings show that CA1 pyramidal neurons and striatal neurons undergo terminal AD induced by OGD or ouabain exposure. A1) In response to 10 minutes of OGD the CA1 neuron initially hyperpolarizes before rapid AD (arrow) to near-zero millivolts from which it does not recover. Inset shows typical voltage responses of a CA1 neuron in response to hyperpolarizing and depolarizing current pulses. A2) CA1 neuron exposed to 10 minutes of 100 µM ouabain. The neuron initially depolarizes and discharges, leading to spike inactivation just prior to a rapid AD-like response (arrow) from which the neuron does not recover. B1) Tonic firing by three striatal neurons in response to a depolarizing current pulse. B2) In response to OGD a striatal neuron slowly depolarizes before undergoing rapid AD to near-zero millivolts. Upon return to control aCSF, the neuron begins to repolarize, but does not recover. B3) In response to 10 minutes of ouabain exposure, a striatal neuron slowly depolarizes before a rapid AD-like response to near-zero millivolts (arrow). Upon return to control aCSF there is no recovery.

The patch pipette was visually placed within layer CA1 and pyramidal somata targeted based on the triangular shape and diameter of 15 to 20 µm. The average resting membrane potential was −63±2.5 mV (Table 1). Mean action potential amplitude and input resistance were 84±13.4 mV and 99±18 MΩ, respectively. The inset in Figure 3A illustrates a typical response of a CA1 neuron to hyperpolarizing and depolarizing current steps. Nine CA1 pyramidal neurons were monitored by whole-cell patch during simulated stroke. Ten minutes of OGD elicited an abrupt AD in CA1 neurons that reached zero millivolts in all cells tested (Fig. 3A1). Mean AD onset time was 343±85 seconds, with an average rate of 3.0±0.9 mV/s during the fast component of the AD. Seven cells responded to OGD with an initial hyperpolarization (*H*, Fig. 3A1). Following 20 minutes recovery in control aCSF the mean membrane potential was only −20±7.4 mV, representing a mere 10% recovery (Table 1). Whole-cell input resistance was too low to accurately measure post-OGD. No new recordings could be acquired in the CA1 stratum pyramidale post-OGD as the neurons were all permanently depolarized. The response to OGD by the nine cells was indistinguishable from 17 other CA1 neurons (Table 1) in our previous study [37] so we did not expand the pool further.

**Table 1:** Whole-cell recording parameters from ‘higher’ CA1 and striatal neurons (n = 36) compared to brainstem neurons (n = 65) responding to 10 minutes of oxygen/glucose deprivation.

Neuron Type	Rmp (mV)	Rmp Post-OGD (mV)	% Rmp Recov.	Max Depol. (mV)	% AP Ampl. Recov.	Rin (MΩ)	% Rin Recov.	AD Onset (sec)
**CA1 pyramidal (n = 9)**	−63±2.5	−6±7.4	10±11.8	Near zero	No recovery	99±17.7	No recovery	343±84
**CA1 pyramidal (n = 17)** [^37^]	−62±3.0	−9±4.6	No recovery	Near zero	No recovery	47±8.4	No recovery	318±78
**Striatal (n = 10)**	−70±3.0	−20±16.9	29±23.6	Near zero	No recovery	55±17.6	No recovery	287±23
**Locus Ceruleus (n = 8)**	−48±2.8	−40±10.1	82±19.4	−4±4.6	83±15.0	219±88	100±11	301±60
**Mesencephalic (n = 14)**	−53±2.3	−50±6.0	93±10.2	−42±11.5	95±8.1	48±41	86±23	301±60
**Dorsal nuc. tractus solitarius (n = 31)**	−48±4.5	−36±11.6	75±23	−10±7.8	73±13.4	785±470	57±30	322±87
**Dorsal motor nucleus of vagus (n = 12)**	−46±1.4	N/A	N/A	−1±1.4	N/A	278	N/A	∼322

All neurons were exposed to 10 minutes OGD except 16 of the 31 dNTS neurons which underwent 15 minutes of OGD. Post-OGD measurements made 15–30 minutes following return to control aCSF. More details of the electrophysiological properties of each neuron as well as the properties of newly acquired brainstem neurons recorded in post-OGD slices can be found at http://hdl.handle.net/1974/7578 [36]. Abbreviations: Rmp, resting membrane potential; Max Depol., maximum depolarization of anoxic depolarization; AP Ampl., action potential amplitude; Rin, whole-cell input resistance. % Rmp recovery was calculated before correcting for a +14 mV junction potential.

Exposing six CA1 neurons to 100 µM to the Na^+^/K^+^ pump inhibitor ouabain for 10 minutes elicited an abrupt AD-like event (arrow Fig. 3A2) that approached zero millivolts, similar to OGD. During the initial response, the CA1 neuron could fire a series of action potentials followed by spike inactivation, before undergoing rapid AD. Otherwise the response was similar to OGD.

#### Striatal Neurons

The patch pipette was visually placed within the striatum and cells targeted based on their diameter of 10 to 15 µm. Spiny projection neurons fire tonically in response to a depolarizing stimulus (Fig. 3B1)[13]. The average resting membrane potential of the 10 cells was −70±3.0 mV. Input resistance and action potential amplitude were 55±18 MΩ and 81±9.1 mV, respectively, as reported in other studies [38].

Ten striatal neurons were monitored during 10 minutes of OGD which evoked an abrupt AD to near-zero millivolts (Fig. 3B2, Table 1). Mean AD onset was 287±23 seconds with an average rate of 3.0±0.8 mV/s during the fast component of the AD. Upon reintroduction of control aCSF, neurons repolarized to −20±16.9 mV, representing a 29% recovery (Table 1) but then deteriorated further. Cell input resistance did not recover. As with CA1 neurons, no action potentials could be evoked post-OGD. As well, no newly acquired recordings could be obtained in striatum, further indicating regional injury.

Exposing 4 striatal neurons to 100 µM ouabain for 10 minutes elicited an abrupt AD-like response that approached zero millivolts (Fig. 3B3). As with OGD, striatal neurons fired no action potentials during the depolarization phase and no substantial recovery occurred.

### Midbrain/Pontine Neuron Electrophysiology

#### Neurons in Locus Ceruleus (LC)

**Figure 4:**
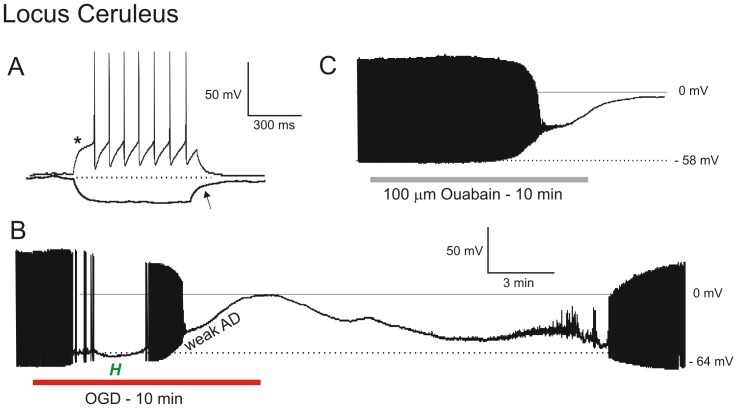
LC neurons resist depolarization in response to OGD or ouabain. A) Typical voltage responses showing characteristic delayed firing (arrowhead) when depolarized from negative holding potentials and a characteristic delay in return to baseline potential (arrow) following a hyperpolarizing pulse. B) Whole-cell recording of an LC neuron responding to 10 minutes of OGD. Initially, the neuron briefly hyperpolarizes as action potentials inactivate before reaching a plateau. It then continues to near-zero millivolts. Upon return to control aCSF, the LC neuron slowly repolarizes. C) LC neuron response to 10 minutes of ouabain exposure. As during OGD, the neuron gradually depolarizes, reaching a brief plateau before continuing to near-zero millivolts. The recording was then lost.

Eight neurons from the LC were whole-cell patched. The pipette was visually placed within the LC and the cells targeted based on their elongated shape and diameter of 20 µm (Henderson et al. 1982). LC neurons were identified by a characteristic delay in firing (A-type K^+^ current) in response to a depolarizing current step from holding potentials below −70 mV (asterisk, Fig. 4A). Following a hyperpolarizing current step, LC neurons also displayed a characteristic delay in return to membrane potential (arrow, Fig. 4A). The average resting membrane potential of the 8 LC neurons tested was −48±2.8 mV. Mean input resistance and action potential amplitude were 219±88 MΩ and 95±6.4 mV, respectively, as reported in other studies [39], [40]. Ten minutes of OGD gradually depolarized spiking LC neurons, reaching −28±3.5 mV (Fig. 4B). After this plateau, LC neurons resisted further depolarization (≤10 mV) for an average duration of 57±21 seconds, before further depolarizing to −4±4.6 mV (Table 1). Mean AD onset was 298±57 seconds as measured in adjacent MES neurons. The average membrane potential of 6 LC neurons that were held following 10 minutes of OGD was −40±10.1 mV, representing 87% recovery (Table 1). Mean input resistance was 229±57 MΩ, an increase that was likely caused by partial pipette block during cell swelling. Action potential amplitude was 79±11 mV (83% recovery).

Exposing four LC neurons to 100 µM ouabain for 10 minutes caused gradual depolarization with slow spike inactivation (Fig. 4C), similar to OGD. Also similarly, the neurons temporarily resisted further depolarization at approximately −25 mV before depolarizing to −2 mV. All 4 neurons were lost during ouabain washout, but their hyperpolarizing trajectories indicated some initial recovery.

#### Neurons in Trigeminal Mesencephalic (MES) Nucleus

**Figure 5:**
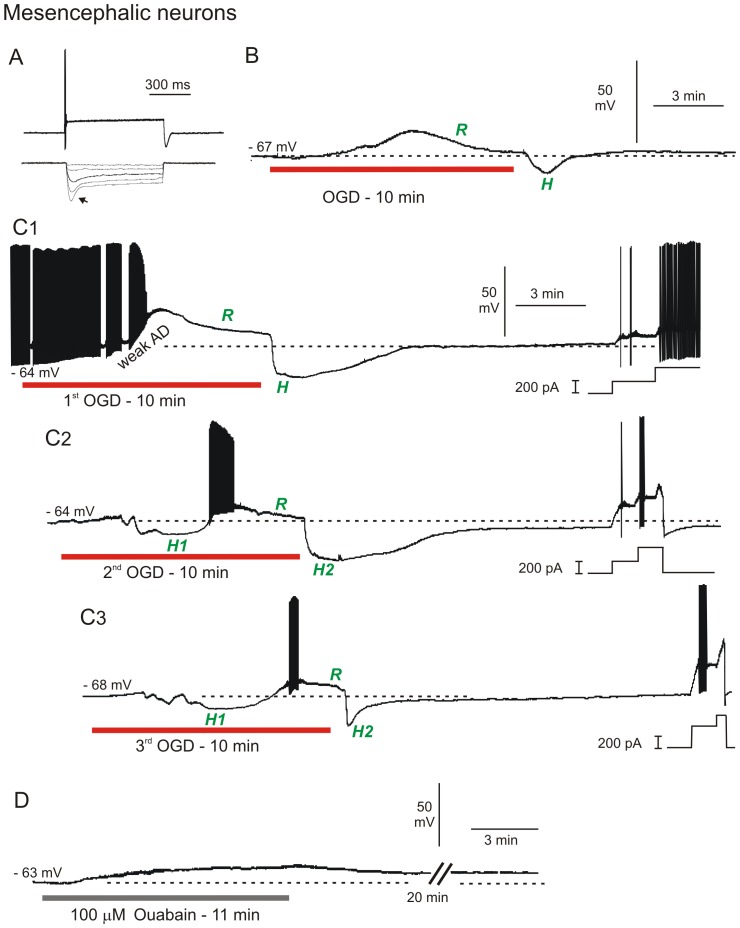
MES neurons strongly resist depolarization in response to OGD or ouabain. A) MES neurons typically show strong spike accommodation in response to a depolarizing current step (top trace) and strong inward rectification (arrow) in response to a hyperpolarizing current step (bottom trace). B) Whole-cell recording of MES neuron in response to 10 minutes of OGD. The neuron weakly depolarizes followed by active repolarization (*R*) during OGD. With return to control aCSF, the neuron displays a hyperpolarizing undershoot (*H*) and returns to near resting potential. C) MES neuron response to three 10-minute OGD exposures. C1) The neuron initially fires spontaneously and inactivates followed by active repolarization during OGD (*R*). With return of control aCSF, the neuron displays a hyperpolarizing undershoot as in B, before regaining its original membrane potential. At the end of the recording, steps of depolarizing current demonstrate ability to discharge. C2) In response to a second OGD period, the MES neuron initially hyperpolarizes (*H1*) before firing action potentials that inactivate as in C1, followed by active repolarization during OGD (*R*). Upon return to control aCSF, the cell hyperpolarizes (*H2*). Incremental current steps (right) show firing and strong accommodation. C3) As above, the neuron initially hyperpolarizes, inactivates and actively repolarizes during OGD, followed by a brief hyperpolarizing undershoot upon return to control aCSF. D) MES neuron response to 10 minutes ouabain exposure. The cell depolarizes but unlike with OGD, there is no repolarization or hyperpolarizing undershoot upon return to control aCSF.

A patch pipette was visually placed within MES nucleus and the cells targeted based on their large diameter of 25 to 50 µm and their round or ellipsoid shape [41]. MES neurons were further identified by strong action potential accommodation and pronounced inward rectification (arrow, Fig. 5A) in response to depolarizing and hyperpolarizing current steps, respectively. The majority of MES neurons were silent at resting membrane potential (Fig. 5A,B), although two fired oscillating bursts of action potentials (Fig. 5C1). The average resting membrane potential of the 14 MES neurons was −53±2.3 mV (Table 1). Mean input resistance and action potential amplitude were 48±41 MΩ and 75±7.1 mV, respectively, as reported in other studies [41], [42].

Exposing 14 MES neurons to 10 minutes of OGD elicited only slight depolarization, reaching a mean of −42±11.5 mV (Fig. 5B) with a mean onset time of 301±60 seconds. The majority of MES neurons were silent but some initially fired spontaneously prior to OGD (Fig. 5C1). Remarkably, all MES neurons actively repolarized during OGD (*R*, Figs. 5B, C1). Upon return to control aCSF, MES neurons often displayed a hyperpolarizing undershoot (*H*, Figs. 5B, C1) before returning to baseline levels. The average resting membrane potential of 12 MES neurons that could be held following 10 minutes of OGD was −50±6.0 mV, a 93% recovery (Table 1). Input resistance and action potential amplitude were 38±24 MΩ (86% recovery) and 72±12.7 mV (95% recovery), respectively. Moreover healthy MES neurons could be readily patched in slices previously exposed to 10 minutes of OGD. Their average resting membrane potential (n = 7) was −52±2.4 mV, representing 99% recovery of membrane potential, compared to MES neurons not previously exposed to OGD. Input resistance and action potential amplitude were 48±23 MΩ (100% recovery) and 73±6.2 mV (97% recovery), respectively.

Some MES neurons were subjected to additional 10 minute OGD exposures (Fig. 5C). In response to the first 10 minute OGD period, a mesencephalic neuron depolarized to −31 mV, with accompanying action potential inactivation (Fig. 5C1). AD onset, measured at the start of depolarization was 315 seconds. Then during OGD, the neuron resisted further depolarization by actively repolarizing. Upon return of control aCSF, the neuron underwent a rapid repolarization with a hyperpolarizing undershoot (*H*) before returning to its original potential. Near the end of the trace, depolarizing current steps demonstrated the cell's ability to fire. The response to a second and third 10 minute OGD period was similar, except the neuron was now silent and hyperpolarized even before depolarization (*H1*, Fig. 5C2,3), before depolarizing to −43 mV with slow spike inactivation. The second AD onset was 117 seconds later than the first OGD exposure (compare Figs. 5C1, 2). Post-OGD the neuron hyperpolarized (*H2*) before the membrane potential returned to near its original resting potential. At the end of the traces, current injection induced a burst of action potentials that accommodated. In response to a third OGD, AD onset occurred 177 seconds later than the first (compare Figs. 5C1, 3) so the cell was becoming more resistant to depolarization with each OGD period.

Exposing three MES neurons to 100 µM ouabain for 10 minutes caused a gradual depolarization, similar to OGD (Fig. 5D). As with OGD, the recorded neuron resisted further depolarization during ouabain exposure, reaching a maximal depolarization of −39 mV. Upon return of control aCSF, membrane potential slowly returned to −44 mV (92% recovery), likely reflecting the slow unbinding and washout of ouabain typical of all neuronal types we recorded. Input resistance and action potential amplitude were 48 MΩ (79% recovery) and 48 mV (80% recovery), respectively. Two other MES neurons could not be maintained for the recovery phase but following 20 minutes of slice recovery in control aCSF, two newly acquired MES neurons were obtained with resting membrane potential, input resistance and action potential amplitude similar to MES neurons not exposed to ouabain. This provided further evidence for MES recovery from ouabain exposure.

### Two photon microscopy confirms OGD-induced injury to CA1, but not to MES neurons

**Figure 6:**
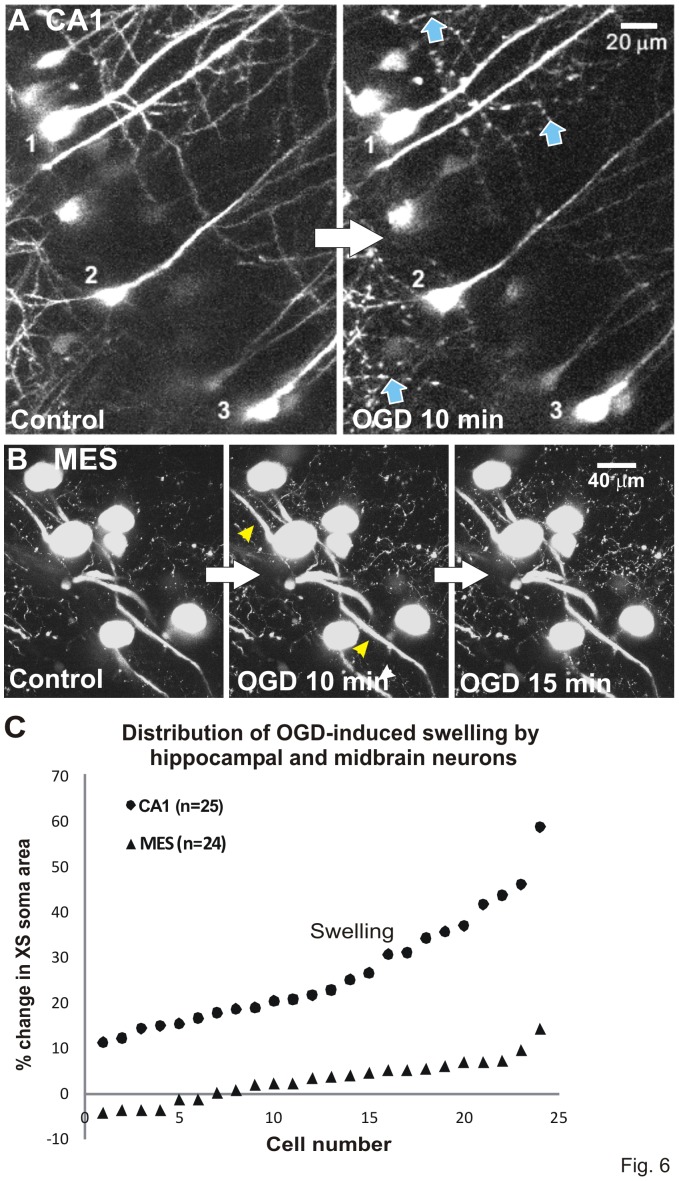
OGD injures CA1 pyramidal neurons but not MES neurons. Volume responses to OGD by YFP-positive neurons monitored in real time with 2-photon laser microscopy. A) Three CA1 pyramidal cell bodies display swelling as their dendrites form beads (arrows). This is an all-or-none response post-AD [30] and was not further quantified. Both responses denote acute damage caused by 10 minutes OGD. There is no recovery (not shown). B) Neuronal cell bodies in the MES nucleus resist swelling after 10 or 15 minutes of OGD. The primary processes display minor swelling by 10 minutes (arrowheads). C) Change in cross-sectional (XS) area of the cell body is measured to determine soma swelling or shrinking in real time. Swelling by ‘upper’ CA1 neurons is pronounced compared to the small range displayed by ‘lower’ MES neurons in response to 10 minutes OGD. Results are similar to [26] comparing ‘upper’ neocortical pyramidal neurons and ‘lower’ hypothalamic magnocellular endocrine neurons.

Change in the cross-sectional (XS) area of the cell body was measured to determine real time swelling of somata in response to 10 minutes of OGD. Immediately post-OGD, CA1 cell bodies swelled and dendrites lost their spines and became beaded (arrows, Fig. 6A). There was no recovery over the next 30 minutes (not shown). This sudden structural disruption requires AD generation and is described in the Introduction. In contrast MES neuronal somata resisted swelling after exposure to 10 or 15 minutes of OGD, with only minor swelling of primary processes (arrowheads, Fig. 6B). Figure 6C shows the distribution of the % change of cross-sectional area for CA1 and MES cell bodies, demonstrating dramatic swelling of CA1 cells in contrast to little or no detectable swelling by MES cells.

### Medullary Neuron Electrophysiology

#### Neurons in Dorsal Nucleus Tractus Solitarius (dNTS)

**Figure 7:**
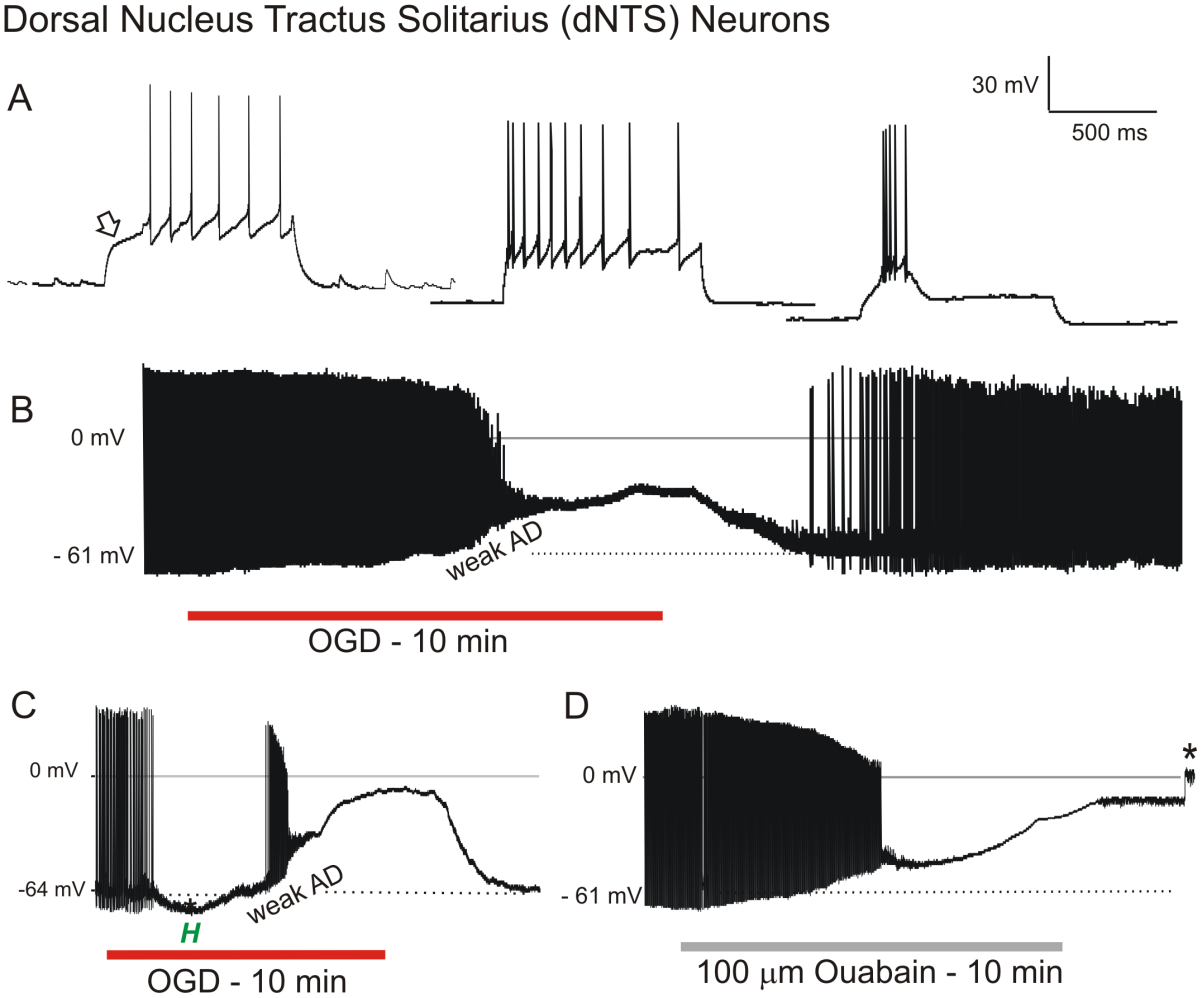
Sensory neurons in brainstem resist depolarization in response to OGD or ouabain. A) Response by three dNTS neurons at resting potential to depolarizing current steps, displaying either delayed firing (arrow, left trace), regular firing (middle trace) or burst firing (right trace). B) In response to 10 minutes of OGD, the dNTS neuron gradually depolarizes with eventual spike inactivation, reaching a plateau that is maintained during OGD. Upon return of control aCSF the neuron repolarizes to near resting potential with spontaneous action potential firing. C) A similar response to OGD as seen in B but with an early hyperpolarization (*H*). D) Response by dNTS neuron to 10 minutes of ouabain exposure. The neuron gradually depolarizes before reaching a plateau similar to the OGD response in C. Upon return to control aCSF the recording is lost (asterisk).

Thirty-one sensory neurons from dNTS of the medulla were whole-cell patched during simulated stroke. The patch pipette was visually placed within the dNTS and the cells targeted based on their diameter of 15 to 20 µm. In response to a depolarizing current step dNTS neurons were further categorized into delayed firing (n = 12, left trace, arrow), regular firing (n = 10, middle trace) or bursting (n = 9, right trace, Fig. 7A) [43]. Rebound depolarization following a hyperpolarizing current step was recorded in most dNTS neurons (not shown). The average resting membrane potential of 31 dNTS neurons was −48±4.5 mV (Table 1). Mean input resistance and action potential amplitude were 785±470 MΩ and 86±10.1 mV, respectively, as reported in other studies [44].

Exposing dNTS neurons to 10 minutes (n = 15) or 15 minutes (n = 16) of OGD, elicited either a gradual depolarization (n = 7, Fig. 7B) often preceded a brief initial hyperpolarization (n = 24, Fig. 7C) as in LC neurons (Figs. 4B). The mean plateau of dNTS neurons was −32±5.6 mV with spike inactivation halting discharge. After reaching a plateau, dNTS neurons resisted further depolarization for an average 45±14 seconds, before depolarizing −10±7.8 mV (Table 1). Mean AD onset was 322±87 seconds. The average membrane potential of 17 dNTS neurons that were maintained following 10 or 15 minutes of OGD was −36±11.6 mV, representing a 75% recovery. Input resistance and action potential amplitude were 676±346 MΩ (57% recovery) and 62±10.5 mV (73% recovery).

Healthy neurons from the dNTS could be readily patched following 10 or 15 minutes of OGD. The average resting membrane potential of 12 dNTS neurons that were newly acquired following 10 or 15 minutes of OGD was −45±3.1 mV, a 95% recovery compared to neurons not previously exposed to OGD. Mean cell input resistance and action potential amplitude were 701±386 MΩ (89% recovery) and 69±13.6 mV (80% recovery), respectively.

Exposing a slice to 100 µM ouabain for 10 minutes elicited gradual depolarization of dNTS neurons (Fig. 7D), similar to OGD. As with OGD, the recorded dNTS neuron resisted depolarization during ouabain exposure, maintaining a brief plateau of −39 mV before depolarizing to −9 mV. Upon return of control aCSF, the recording was lost (asterisk, Fig. 7D).

### Neurons in Dorsal Motor Vagal (DMV) Nucleus

**Figure 8:**
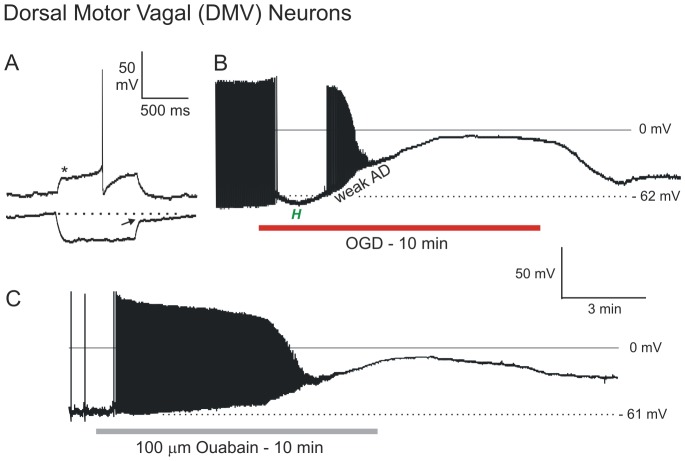
Motor neurons in brainstem resist depolarization in response to OGD or ouabain. A) Typical response of a DMV neuron to current pulse injection. When depolarized from negative holding potentials, DMV neurons display characteristic delayed firing (asterisk) as well as a delay in return to baseline(arrow) following a hyperpolarizing current step. B) During OGD, a DMV neuron initially hyperpolarizes before depolarizing with slow spike inactivation to −5 millivolts. Back in control aCSF the neuron repolarizes. C) DMV neuron response to 10 minutes of ouabain exposure. The neuron gradually depolarizes to −10 mV. Upon return to control aCSF the neuron starts repolarizing but the recording is lost (not shown).

The patch pipette was visually placed within the DMV nucleus and the cells targeted based on their ellipsoid shape and diameter of ∼20 µm [45]. DMV neurons displayed a characteristic delay in firing (generated by an A-type K^+^ channel) in response to a depolarizing current step from holding potentials below −70 mV (asterisk, Fig. 8A). In response to a hyperpolarizing current step, DMV neurons also displayed a characteristic delay in return to baseline potential (arrow). The average resting membrane potential of the 12 DMV neurons tested was −46±1.4 mV. Mean input resistance and action potential amplitude were 265±75 MΩ and 94±5.3 mV, respectively, as earlier reported [46].

Twelve neurons from the DMV nucleus were recorded during simulated stroke. OGD elicited either a gradual depolarization (n = 5, not shown) or a brief hyperpolarization (n = 7, Fig. 8B) that had a mean duration of 154±44 seconds before reaching a plateau of −28±2.1 mV. DMV neurons that hyperpolarized in response to OGD had a delay to onset of the plateau depolarization by 93 seconds in comparison to gradually depolarizing DMV neurons. After reaching the plateau, DMV neurons resisted further depolarization (≤10 mV) for 76±30 seconds, before depolarizing to −1±1.4 mV. Mean AD onset was not measured in DMV neurons, but was similar to adjacent neurons of the solitary tract, which underwent AD at 322±87 seconds (Table 1). With return to control aCSF, DMV neurons began repolarizing but recordings could not be maintained. However newly acquired DMV neurons could be readily recorded in slices recovered from 10 min OGD. The average resting potential of 10 new DMV neurons was −45±2.7 mV, representing 98% recovery compared to neurons not previously exposed to OGD. Mean input resistance and action potential amplitude were 265±75 MΩ (95% recovery) and 69±9.6 mV (73% recovery), respectively.

Exposing DMV neurons to 100 µM ouabain for 10 minutes elicited a gradual depolarization (Fig. 8C), maintaining a brief plateau of −23 mV, before further depolarization to −7 mV. Slow repolarization to −34 mV followed 20 minutes of control aCSF.

**Figure 9:**
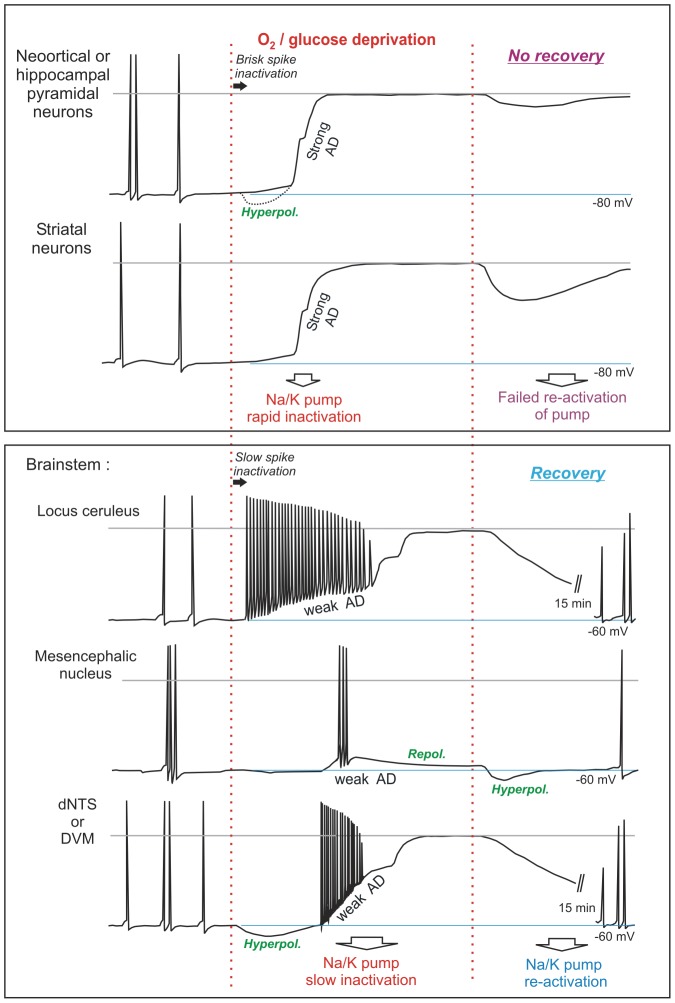
Summary of neuronal responses to 10 to 15 minutes of OGD which are distinct for each neuron type. CA1 pyramidal cells and striatal neurons undergo spike inactivation that is independent of depolarization as well as rapid AD which quickly approaches zero millivolts. These neurons do not recover in brain slices. In contrast, 4 types of brainstem neuron undergo both gradual spike inactivation and gradual AD. These neurons substantially repolarize post-OGD. Of note, MES neurons maintain their resting potential in the face of OGD. Note that hippocampal and striatal neurons are similar to neocortical and thalamic neurons; the brainstem neurons are similar to hypothalamic neurons [24], [26]. We propose that the properties noted above are most easily explained by brainstem and hypothalamic neurons possessing more resilient versions of the Na^+^/K^+^ pump than higher neurons.

Characteristic responses to OGD by the different neuronal types examined above are summarized in Figure 9. Typical of higher neurons, pyramidal and striatal neurons rapidly and completely depolarize during OGD and cannot recover. Brainstem neurons display less polarized resting membrane potentials (Table 1) yet better resist OGD with a delayed and weak depolarization, followed by repolarization and recovery post-OGD.

## Discussion

Whether evoked by traumatic brain injury, near-drowning, cardiac arrest or pulmonary failure global ischemia deprives the *entire* brain of oxygen and glucose. Notably there is no evidence for a preferred or selective regional reperfusion of brainstem under any of these diverse situations. Also there are no data indicating that higher brain regions consume more energy per unit volume than lower regions, that a cortical neuron requires more energy than a brainstem neuron or that neurons from higher and lower regions differ in their ion channel composition. Yet the outcome from global ischemia is often diffuse damage to the higher brain with sparing of the hypothalamus and brainstem. As a result, over several weeks many afflicted patients transition from coma to a persistent vegetative state [3]–[5], [12], [13], [47]. Neocortex, striatum, hippocampus and cerebellar cortex are particularly vulnerable to ischemia [17], [48]–[51]. In the current study using brain slices, we document a terminal AD in these higher regions but a weak and recoverable AD in all four brainstem nuclei recorded.

Several studies using LT imaging in live cortical slices exposed to OGD show that, as the AD front passes by a recording electrode, there is a sudden mass depolarization of neurons. The cells do not repolarize based on non-reversible light scattering and lost evoked excitability [19], [20], [24], [26], [33], [34]. Here we show that milder events can be observed in brainstem in the same coronal slices that support strong AD in hippocampus or cerebellar cortex. Dorsal brainstem nuclei display mini-fronts of AD but recover to baseline with nominal light scattering, indicating minimal neuronal damage. AD propagation is significantly slower and less damaging than higher gray regions with the exception of the superficial superior colliculus (see end of Discussion). These optical data suggest that the brainstem is comparatively protected from OGD. We investigated further using whole-patch recording.

OGD eliminates the evoked field potential in neocortical and hippocampal slices and this requires that the AD propagates through the recorded region [20], [26]. LT imaging reveals a similar vulnerability of striatal neurons in slices [13], [21], [34], [37] as also recorded in vivo in the ischemic core [17], [48], [49]. The current study demonstrates the high sensitivity of pyramidal neurons to ischemia, cells that have been the prototype for CNS neurons under ischemic stress. Clearly this assumption cannot be extended to brainstem neurons which are slow to spike-inactivate and only depolarize gradually (below). Importantly, strong AD elicits vasoconstriction in neocortex (inverse neurovascular coupling) which exacerbates ischemic injury [16]–[18]. Whether weaker AD evokes less vasoconstriction in the brainstem is an important and open question.

Neurons of the midbrain-pons easily survived 10 minutes of OGD. Locus ceruleus neurons and the mesencephalic neurons of the trigeminal nucleus recovered 80 to 100% of their membrane potential, input resistance and action potential amplitude. Moreover post-OGD, newly acquired neurons could be readily obtained in LC and MES nuclei. Said neurons displayed membrane potentials, whole cell input resistances and action potential amplitudes similar to neurons not previously exposed to OGD [36]. Some MES neurons even recovered from multiple OGD exposures. MES neurons are primary sensory neurons, similar to dorsal root ganglia neurons in the periphery. They are derived from neural crest cells, but migrate into the CNS [52]. They are the most resistant to OGD and ouabain of the neurons we have tested, showing dorsal root ganglia-like resiliency described by others [53]–[55]. The hyperpolarizations we recorded during/immediately after OGD are blocked by ouabain so they are likely generated as OGD up-regulates the Na^+^/K^+^ pump, a likely scenario given that the pump normally operates at only 50% capacity [56].

Medullary neurons of dNTS and DMV [57] also survived the OGD period that killed pyramidal and striatal neurons. DNTS cells recovered >70% of their mean membrane potential and action potential amplitude post-OGD. Newly patched neurons could be readily obtained in both DMV and dNTS that had recovered from OGD [36].

So in contrast to hippocampal and striatal neurons which are quickly silenced by spike inactivation and depolarization, brainstem neurons depolarize slowly and are able to fire during both OGD onset and recovery. This gradual depolarization may activate brainstem neurons in cardio-respiratory centers that could aid a subject during hypoxia [57] or ischemia. Indeed, the LC projects to a number of brain regions involved in circulatory regulation including hypothalamus, nucleus of the solitary tract, nucleus ambiguous and dorsal motor nucleus of the vagus [58]. This neuronal resilience would help maintain vital functions during metabolic stress.

### The crucial role of Na^+^/K^+^-ATPase Pump

In each neuronal type tested, exposure to 100 µM ouabain evoked depolarization similar to that elicited by OGD but lacking the hyper- or repolarizations seen with OGD. In pyramidal neurons, ouabain induced rapid depolarization to near zero millivolts [24], [26], [37], [59] whereas brainstem neurons only gradually depolarized and recovered. So as is consistently observed with intracellular recording [24] and imaging [19] ouabain exposure re-iterates the OGD response by inducing Na^+^/K^+^ pump failure. Ouabain recovery is slow because it is tightly bound to the pump. The α2 and α3 isoform constituents of the ATPase molecule reduces vulnerability to ischemia compared to α1 versions [60], [61] and we have mined data (unpublished) from the Allen Brain Bank [62] indicating that these resilient versions are proportionally most highly expressed in the hypothalamus and brainstem.

The molecular events linking Na^+^/K^+^ pump failure to mass neuronal depolarization has remained a mystery. The anoxic (or ‘ischemic’) depolarization is not simply the result of a slow run-down of the pump. Rather AD is driven by a formidable inward Na^+^ current with K^+^ efflux that accumulates extracellularly [21], [63], [64]. Yet blockers of standard Na^+^ and K^+^ channels only delay AD onset (ibid) and glutamate receptor activation is not required for AD except in cerebellar gray [14], [19], [21], [34], [64]. AD block requires a concentrated cocktail of ion-gated and ligand-gated channel antagonists that electrically silences the tissue [65]–[67]. The pannexin1 hemichannel [68] opens later during AD and appears not to be the primary conduit driving OGD-induced AD [65], [69] or spreading depression [70].

An unexplored possibility is that the Na^+^/K^+^ transporter itself is converted to an open channel that generates AD under ischemic conditions [71]. At nanomolar concentrations the marine poison palytoxin converts the ATPase molecule into an open cationic channel [72]. The resulting pore is wide enough to pass organic cations of <195 Da [73]. Held open, millions of Na^+^ and K^+^ ions/sec are conducted per channel, whereas normally only ∼100 ions/sec are transported [74]. Given that there are thousands of Na^+^/K^+^ ATPase molecules in the plasma membrane of a single neuron, the resultant propagating current would be formidable, as evidenced by the AD-like response evoked by only 1 nM palytoxin [71].

In summary, overriding the Na^+^/K^+^ pump using OGD, ouabain or palytoxin evokes steep and robust depolarization of neurons in higher, but not lower gray matter. Pump efficiency during ischemic stress is likely a major factor in generating strong or weak spreading depolarizations by the brain. Understanding how brainstem neurons resist ischemia (perhaps by expressing pump isoforms that better function under OGD) can help us understand why higher neurons are so vulnerable to ischemia and thus reveal new targets to promote protection.

The question arises as to why the rostral brain is programmed for ischemic vulnerability when ideally all neurons should be as resilient as possible. We propose that robust spreading depolarization is not simply a pathological mechanism, but is an active ‘shutdown’ process that has evolved in response to reduced blood flow caused by head trauma and has conferred a survival advantage as vertebrates have evolved [75]. For example complete depolarization of a traumatized cortical area is preferable to a partial depolarization which can generate spastic or seizure activity. Upon head impact, immediately falling to the ground in an unconscious state reduces attention to the vulnerable individual, lessens the chance of further injury and lowers the metabolic rate, as well as reducing bleeding by lowering blood pressure and heart rate. A similar shutdown of brainstem nuclei would be instantly fatal.

## Supporting Information

Movie S1 **Inhibiting the Na^+^/K^+^ pump with OGD evokes AD in a live parasagittal slice.** AD arises initially in the hippocampal formation, coursing through CA3, CA1 and then dentate gyrus (pseudocolored blue-yellow denoting cell swelling) at ∼5 minutes of OGD exposure. AD next arises in frontal neocortex at ∼6 minutes and striatum at about 9 minutes. Upon return to control saline at 10 minutes these regions show ever increasing light scatter (purple pseudocoloring) denoting neuronal damage. There is no recovery over the next 20 minutes. Striatum displays minimal damage likely because AD is generated late in the OGD period.
(MP4)

Movie S2 **Blocking the Na^+^/K^+^ pump with ouabain evokes an AD-like response in a live coronal slice.** Image sequence showing an AD front propagating at 2–3 mm/minute through neocortical gray and then through striatum. The wave of elevated LT is induced at about 5 minutes of bath exposure to 100 µM ouabain. By 6 minutes the LT reduction (pseudocolored purple denoting neuronal injury) slowly increases in the wake of AD. By the end of video, ouabain (applied for 10 minutes) has been washed out for 30 minutes with no recovery. This LT sequence is similar to responses evoked by OGD (Fig. 1A; Movie S1) or palytoxin (Fig. 1B).
(MP4)
